# A case of surgically-associated anti GQ1b antibody syndrome accompanied by saccadic ping pong gaze

**DOI:** 10.1186/s12883-019-1258-x

**Published:** 2019-02-19

**Authors:** Jingzhe Han, Yanan Xie, Haiyan Yan, Yuecheng Cao, HongmeiWang, Duanhua Cao

**Affiliations:** 1 Department of Neurology, Harrison International Peace Hospital, 2 Renmin Zhong Road, Taocheng District, Hengshui, 050000 Hebei China; 2 Department of Angiocardiopathy, The Second Hospital of Hebei Medical University, Shijiazhuang, Hebei China; 3 Department of Cardiac surgery, Harrison International Peace Hospital, Hengshui, 050000 Hebei China

**Keywords:** Periodic alternating ping-pong gaze, PPG, Anti GQ1b antibody syndrome, BBE

## Abstract

**Background:**

Periodic alternating ping-pong gaze (PPG) is a rare disease with few reports. To our knowledge, there was no report on anti GQ1b antibody syndrome accompanied by PPG. This paper reported a case of anti GQ1b antibody syndrome with Bickerstaff’s Encephalitis (BBE) overlapping classic Guillain-Barre Syndrome (GBS) after aortic valve replacement, accompanied by an excessive PPG in the course of diagnosis and treatment, this was indeed rarely.

**Case presentation:**

A 55-year-old male patient was admitted to our hospital with intermittent chest tightness for 3 months, and his condition has worsened in the past 10 days. Aortic valve replacement was performed because of the existence of the moderate and severe stenosis of aortic valve. Horizontal movement of the eyeball was involuntarily slow. The eyeball hovered and returned from one side to the other horizontally for 3–4 s per cycle. In combination with the patient’s typical clinical and laboratory tests, the final diagnosis was anti GQ1b antibody syndrome BBE combined with GBS, accompanied by saccadic ping pong gaze. Intravenous immunoglobulin (0.4 g/kg) was given for immunomodulation, methylprednisolone (1000 mg) therapy and symptomatic treatment were performed in the patient.

**Conclusions:**

The patients were discharged from hospital on the thirtieth day because of economic reasons. After 6 months of follow up, the patients left behind a lack of fluency in speech and limb mobility, but the basic life can be taken care of by himself.

**Electronic supplementary material:**

The online version of this article (10.1186/s12883-019-1258-x) contains supplementary material, which is available to authorized users.

## Background

Anti-GQ1b antibodies were induced by microbial infections such as Campylobacter jejuni and Haemophilus influenzae. Then GQ1b antibodies were combined with GQ1b antigens located in oculomotor nerve, trochlear nerve, abducent nerve, muscle spindle and brainstem, which resulted in spectrum of autoimmune diseases in central and peripheral nervous system diseases, this is the anti-GQ1b antibody syndrome firstly proposed by Odaka et al. in 2001 [1]. According to different clinical manifestations, anti GQ1b antibody syndrome [1] can be divided into the following types: Miller Fisher Syndrome (MFS), Bickerstaff ‘s Encephalitis (BBE), ataxia Guillain-Barre Syndrome (GBS), acute extraocular muscle paralysis, acute throat muscle paralysis and different overlapping types, such as MFS overlapping GBS, BBE overlapping GBS, etc. [2]. Surgical and trauma related GBS have been reported, but most of them are related to classic GBS [3].

Periodic alternating ping-pong gaze (PPG) was firstly described by Fisher in 1967, which was defined as a continuous eye movement, characterized by conjugate movements from one side to the other in a period of 3 to 7 s [4]. PPG can be also related to stroke [5] and metabolic causes [6] has been reported, the latest reports are related to drug toxicity [7, 8]. To our knowledge, there was no report on anti GQ1b antibody syndrome accompanied by PPG.

This paper reported a case of anti GQ1b antibody syndrome with BBE overlapping classic GBS after aortic valve replacement, accompanied by an excessive PPG in the course of diagnosis and treatment, this was indeed rarely.

## Case report

A 55-year-old male patient was admitted to our hospital with intermittent chest tightness for 3 months, and his condition has worsened in the past 10 days. Physical examination showed left enlargement of cardiac boundary, and the systolic murmur (4/6 level) could be heard in the auscultation area of the aortic valve. Cardiac color Doppler ultrasound showed aortic valve calcification with moderate to severe stenosis. Sixth days after admission, aortic valve replacement was performed in the patient successfully without ischemia and hypoxia.

**Fig. 1:**
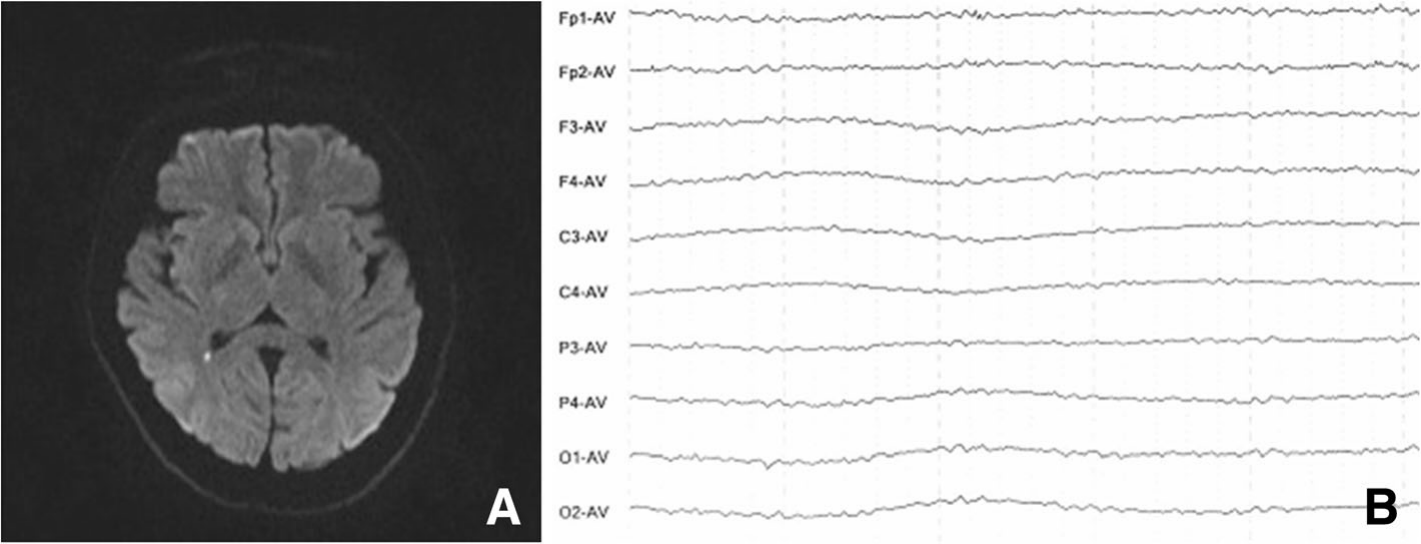
**a** Magnetic resonance DWI showed a small DWI high signal in the right side of the lateral ventricle, acute cerebral infarction was considered. **b** Electroencephalogram (EEG) showed that brain waves were generally 6-8 Hz waves, and were bilateral symmetrical, and had a voltage of 10–25 microvolts

On the seventh days of admission, the patient’s consciousness was clear, his limbs were moving well, and he can communicate with his family simply. On the 11th day of admission, the patient was emotionally agitated, with speech disorder, accompanied by eating cough and diplopia. Dysarthria and ptosis in both eyelids were existed. Both eyes abduct was limited. Bilateral frontal lines and nasolabial sulcus remained unchanged. The muscle strength of the extremities was grade 4+, but the tendon reflex of both lower limbs was decreased. Serum anti-GQ1b antibody test was positive, then postoperative concurrent GBS was considered. Intravenous human immunoglobulin (0.4 g/kg) was given for immunomodulation, methylprednisolone ((Manufacturing Belgium NV, 1000 mg) therapy and symptomatic treatment were performed. On the thirteenth day of admission, the patient’s consciousness turned to sleepiness, and his breathing and heart rate were stable, and the Glasgow Coma Scale/Score (GCS) was 12. Magnetic Resonance Imaging (MRI) + Magnetic Resonance Angiography (MRA) showed small DWI high signal near the posterior corner of right ventricle, acute cerebral infarction was considered (Fig. 1a). On the 16th day of admission, the patient presented with deep coma, poor cough reflex and more sputum. He was given tracheotomy with GCS score of 5 points. The diameter of bilateral pupils is 5 mm, which is slow to reflect light. and the ptosis of both eyelids was existed. Horizontal movement of the eyeball was involuntarily slow. The eyeball hovered and returned from one side to the other horizontally for 3–4 s per cycle (Periodic alternating ping-pong gaze, Additional file 1: Video 1). The muscle strength of the extremities was grade 0, the tendon reflex of the extremities disappeared and the muscle tension was low. On the 18th day, the video EEG (electroencephalograph) showed that the patient was in a coma, and the brain waves were in the general 6-8 Hz wave and bilateral symmetry, and the voltage was 10–25 microvolts, no abnormal electroencephalogram activity was observed in all leads with 6–8 HZ wave (Fig. 1b).

The electromyography of the extremities showed that the motor amplitude of the common peroneal nerve of the left lower extremity decreased, the conduction velocity of the right peroneal nerve slowed down, and the conduction velocity of the superficial peroneal nerve of the right lower extremity slowed down. The occurrence rate of F wave of bilateral median nerve, right ulnar nerve, bilateral common peroneal nerve and left tibial nerve was low. On the 19th days, the patients turned into a shallow coma, PPG disappeared and the GCS score was grade 7. The lumbar puncture treatment showed that the pressure was 120 mmH2O, the cerebro-spinal fluid (CSF) protein was 86 mg/dl associated with pleocytosis (8 cells/ml). The patient gradually turned to get consciousness, and the GCS score was 15 on the 25th days after admission. Dysarthria, slow reflex of pharynx and ptosis of the double eyelids existed. Bilateral pupils were large and equal circles, which were sensitive to light reflection. Both eyes were limited in adduction and abduction. Both frontal lines and nasolabial sulcus became shallow. The muscle strength of the extremities was 2 levels, the muscle tension was low, and bilateral tendon reflex was weakened.

Combining with the patient’s typical clinical and laboratory tests, the final diagnosis was anti GQ1b antibody syndrome BBE combined with GBS, accompanied by periodic alternating ping-pong gaze. The patients were discharged from hospital on the thirtieth day because of economic reasons. After 6 months of follow up, the patients left behind a lack of fluency in speech and limb mobility, but the basic life can be taken care of by himself. Physical examination showed clear mental reaction and mild dysarthria. No blepharoptosis. Bilateral pupils are large and equal circles, which are sensitive to light reflection. The eyeball moves freely in all directions without nystagmus. Bilateral frontal lines and nasolabial sulcus remained unchanged. The strength of limbs was grade 4 +. Decreased tendon reflex in extremities. The bilateral finger-nose and heel-knee-tibia tests were not (mildly) accurate. Double Pasteur sign was negative.

## Discussion and conclusions

The main clinical manifestations of BBE [9] include acute ophthalmoplegia, ataxia and progressive confusion of consciousness content, decline of level of consciousness, and even drowsiness, coma, and acute limb weakness. BBE is a variant of Guillain Barré syndrome, which is attributed to anti GQ1b antibody syndrome, because of the high positive rate of anti GQ1b antibody in serum. Some BBE patients may combine with acute limb weakness, which is BBE overlapping classic GBS, also belongs to anti GQ1b syndrome. In this study, the acute onset of the elderly male patient was characterized by ophthalmoplegia, progressive worsening confusion of consciousness content, decreased consciousness level and limb weakness, which met the clinical diagnostic criteria of BBE combined with GBS. Combining with the patient’s cerebrospinal fluid protein-cell separation, electromyographic neurogenic damage (showed in electromyography) and positive serum anti-GQ1b antibody, the final diagnosis was anti GQ1b antibody syndrome BBE combined with GBS.

Most patients with anti GQ1b antibody syndrome have a history of precursor infection, such as upper respiratory tract infection, diarrhea and so on. There was no evidence of a history of precursor infection before and after surgery in this patient, suggested that there might be a non-infectious inducement. It was reported that trauma and stress after cardiac surgery, lumbar surgery and abdominal surgery can lead to the occurrence of classic GBS [3]. The complications of BBE and GBS after aortic valve replacement suggested that there may be a common non-infectious inducement of BBE and GBS. It was speculated that the pathogenesis of BBE is also the imbalance of immune system induced by surgical stress. The treatment of this patient mainly refers to the GBS treatment, and the overall prognosis was good. The patient was treated with intravenous immunoglobulin combined with large dose of methylprednisolone. 6 months after followed up, the prognosis was good.

PPG is a rare eye movement abnormality with smooth brain waves, but saccadic form has been described. PPG is often associated with coma, and is associated with diffuse brain injury such as bilateral cerebral hemisphere infarction, hepatic encephalopathy, and drug poisoning, suggesting poor prognosis [10]. In addition, crura cerebri and mesencephalic tegmentum damage also leads to PPG [11, 12]. A proposed mechanism for PPG has been disconnection of the cerebrum from the horizontal gaze centers in the brainstem. Anti-GQ1b antibodies were induced by Campylobacter jejuni and Haemophilus influenzae, and combined with GQ1b antigens of oculomotor nerve, trochlear nerve, abductor nerve, muscle spindle of extremities and brainstem, resulting in central and peripheral nervous system involvement, manifested as extraocular muscle paralysis, ataxia and disturbance of consciousness. The possible mechanism of PPG and coma in this patient is that Anti-GQ1b antibody binds to GQ1b antigen located in the brainstem (reticular structure, midbrain), inhibits the reticular structure of the brainstem, directly blockades the connection between the midbrain foot and the horizontal gaze centers, and produces PPG. But at the same time, oculomotor nerve, trochlear nerve and abductor nerve all contain GQ1b antigen, and most of them are combined with ophthalmoplegia, so ophthalmoplegia can cover up the appearance of PPG, therefore, PPG in this patient was only temporary and lasted for a short time. PPG in the course of disease of our patient was considered to be related to mesencephalic tegmentum damage caused by antigen antibody reaction. In this patient, bilateral eye dissymmetry and not fully abduction of the eyes during ping-pong gaze may be associated with ophthalmoplegia. In addition, low-amplitude coma is a prominent feature of this patient, which is related to the damage of the reticular structure of the midbrain and the superior pons [13]. Therefore, we use EEG results to illustrate it, which may provide some value for the diagnosis of this patient.

Some scholars thought that the tegmental involvement of mesencephalon is a characteristic of BBE [14]. About 1/3 of the patients showed abnormal signals in the upper part of the midbrain, cerebellum and thalamus, which could be relieved or progressed with the course of the disease. During the course of the disease, the patient underwent several head CT examinations, but no characteristic changes were found. Although the patient underwent a head MRI examination, no typical BBE features were found, considering may be related to the timing of MRI examination.

Ping-pong gaze in coma in most cases indicate persistent hemispheric damage, we report a patient with a good outcome, which is related to the etiology. Because anti-GQlb antibody syndrome is a disease with good prognosis, some patients can recover completely without treatment. Only patients with epilepsy, pulmonary edema and consciousness disorder caused by brain stem damage may have a risk of death. In addition, early use of plasma exchange or IVIG treatment for these patients can also significantly improve the prognosis.

The possibility of anti GQ1b antibody syndrome should be considered in the occurrence of trauma and post-operative limb weakness and confusion. It is necessary to analyze various etiologies of PPG comprehensively and avoid misdiagnosis.

## Additional file

Additional file 1: Video 1. The eyeball of the patient hovered and returned from one side to the other horizontally for 3–4 s per cycle (Periodic alternating ping-pong gaze was confirmed). (MP4 3046 kb)

## Abbreviations

BBE: Bickerstaff’s Encephalitis
CSF: Cerebro-spinal fluid
DWI: Diffusion weighted imaging
EEG: Electroencephalograph
GBS: Guillain-Barre Syndrome
GCS: Glasgow Coma Scale/Score
MFS: Miller Fisher Syndrome
MRA: Magnetic Resonance Angiography
MRI: Magnetic Resonance Imaging
PPG: Periodic alternating ping-pong gaze
